# Does temporary mechanical circulatory support with Impella 5.5 induce de novo human leukocyte antigen antibodies production in heart transplantation candidates?

**DOI:** 10.1016/j.jhlto.2024.100072

**Published:** 2024-02-16

**Authors:** Amit Alam, Johanna S. van Zyl, Staci McKean, Ahmad B. Abdelrehim, Hira I. Shakoor, Dana Farsakh, Aayla K. Jamil, Joost Felius, Medhat Askar, Shelley A. Hall

**Affiliations:** a Division of Cardiology, New York University Langone Health, New York, New York; b Baylor Scott and White Research Institute, Baylor Scott and White Health, Dallas, Texas; c Baylor University Medical Center, Baylor Scott and White Health, Dallas, Texas; d Qatar University Health and Department of Immunology, College of Medicine, Qatar University, Doha, Qatar

**Keywords:** de novo antihuman leukocyte antigen, Impella, Impella 5.5, sensitization, temporary mechanical circulatory support

## Abstract

**Background:**

Little is known about de novo human luekocyte antigen (HLA) antibody development with Impella 5.5 temporary mechanical circulatory assist support and downstream effects following heart transplantation in the new heart allocation system.

**Methods:**

Thirteen Impella and 17 control patients without device support were prospectively enrolled between December 2020 and June 2022. HLA antibodies with calculated panel reactive antibodies (cPRA) were assessed pre and postdevice implantation and within 1-year postheart transplantation.

**Results:**

Baseline prevalence of HLA antibodies and median cPRA were similar between groups. Patients in the study arm were on Impella support for a median of 7 days. No significant differences in HLA antibodies were observed postdevice or postheart transplant. One patient in the Impella arm developed rejection and required treatment. One Impella patient died due to infection and 1 control patient died due to primary graft dysfunction.

**Conclusions:**

Short-term use of Impella 5.5 in the new heart allocation system does not appear to increase risk of de novo HLA antibody development. Further studies are needed to validate these preliminary findings.

## Background

Temporary mechanical circulatory support (tMCS) utilization has drastically increased for bridge to transplantation from 10% to 41% since the 2018 United Network for Organ Sharing donor heart allocation system revision in the United States.^1^ Besides mechanical complications, such as bleeding and ischemia that may occur from the placement of these devices, prolonged support may pose several immunological challenges due to increased immune cell activation with development of de novo human leukocyte antigen (HLA) allosensitization.^2^, ^3^

Allosensitization with durable left ventricular assist devices has been reported; however, similar associations in patients with tMCS, namely the Impella 5.5 (Abiomed, Danvers, MA), have not been reported in prospective studies to date. The HLA allosensitization risk of the Impella 5.5 and potential consequences in patients receiving orthotopic heart transplantation (HT) remain unclear. We have previously reported a case of Impella 5.5 patient who developed a significant rise in the calculated panel reactive antibodies (cPRA) without any other sensitizing events.^3^ Additionally, both durable and tMCS use have been speculated to be associated with sensitization based on small retrospective studies and reviews.^2^, ^4^, ^5^, ^6^, ^7^ This supports the hypothesis that recipients of the Impella 5.5 could develop de novo HLA antibodies. Herein, we present the first prospective analysis to investigate whether patients with Impella 5.5 are at increased risk to develop de novo HLA antibodies, graft dysfunction, and rejection.

## Methods

This is a prospective study of HLA antibodies in heart transplant patients who provided written informed consent. The study was approved by the Institutional Review Board of Baylor Scott and White Research Institute, and all patients provided written informed consent. Principles of the Declaration of Helsinki formulated by the World Medical Association, the Declaration of Istanbul, and of the ISHLT ethical statement have been adhered to.

Serum samples from 13 patients who received Impella 5.5 were obtained prior to device insertion, weekly leading to orthotopic heart transplantation, then per standard protocol post-orthotopic heart transplantation until 1-year post-transplant. The control group was non-tMCS patients with ≥2 pretransplant panel reactive antibody tests <8 months prior to transplantation. HLA antibody testing was performed using commercial Luminex-based single antigen bead assays (LABScreen SAB kits; One Lambda, Canoga Park, CA). All sera were treated with EDTA prior to testing. Patients who received tMCS other than Impella 5.5 devices or durable left ventricular assist device (LVAD) support prior to transplantation were excluded. The institutional anticoagulation protocols for Impella patients were as per manufacturer recommendations.

The primary objective was evaluating whether tMCS devices are associated with increased de novo development of HLA antibodies. A similar question was considered in LVAD patients, where a significant difference in pre- to post-cPRA (median [quartiles]: 16 [0-41] vs 0 [0]; *p* < 0.0001) was found comparing LVAD patients to control patients.^8^ Assuming the same difference of 16% as reported in the LVAD trial^8^ for mean cPRA with a mean ± standard deviation of 16 ± 16% and 0 ± 0.5% in the study and control arms, respectively, at least 13 subjects were needed per group to allow at least 80% power at a 5% significance level to detect a difference between groups using a Wilcoxon rank sum test. Sample size estimates were obtained using Power Analysis and Sample Size 2021 software using the Wilcoxon rank-sum test simulation.

Peak HLA measures were compared post-Impella and <1-year post-transplant. Baseline HLA measurements are defined as lowest cPRA <1-month pre-Impella implant or <3-months pretransplant for controls. Peak HLA measures postdevice implant are defined as maximum cPRA between Impella implant and transplant or maximum cPRA pretransplant for control patients. Peak HLAs post-transplant is defined as maximum cPRA <1-year post-transplant. Post-transplant donor-specific antibodies (DSA) with mean fluorescence intensities (MFI) of at least 1,000 were collected up to 1 year and strength classified according to the MFI as low (1,000-4,000), moderate (4,000-9,999), and high (>10,000).

Patient characteristics are reported by groups using medians [interquartile range (IQR): Q1-Q3] that are appropriate for skewed distributions and not affected by outliers and counts (%) for categorical variables. Comparisons between groups were performed using nonparametric Wilcoxon rank sum tests that do not require the assumption of normality for continuous variables and chi-square or Fisher’s exact tests (if expected cell counts ≤5) for categorical variables. An adjusted analysis for change in cPRA from baseline was done using analysis of variance adjusting for sex, infection requiring intravenous antibiotics or hospital admission, treated cytomegalovirus viremia, and baseline cPRA as potential confounding factors. Prior pregnancy was considered as a factor in the model, but was collinear with sex given that all female patients had prior pregnancies, and thus did not allow distinguishing between female sex and prior pregnancy as risk factors. Statistical analyses were performed in R and significance assessed using 2-sided *p*-values <0.05.

## Results

### Baseline characteristics

**Figure 1:**
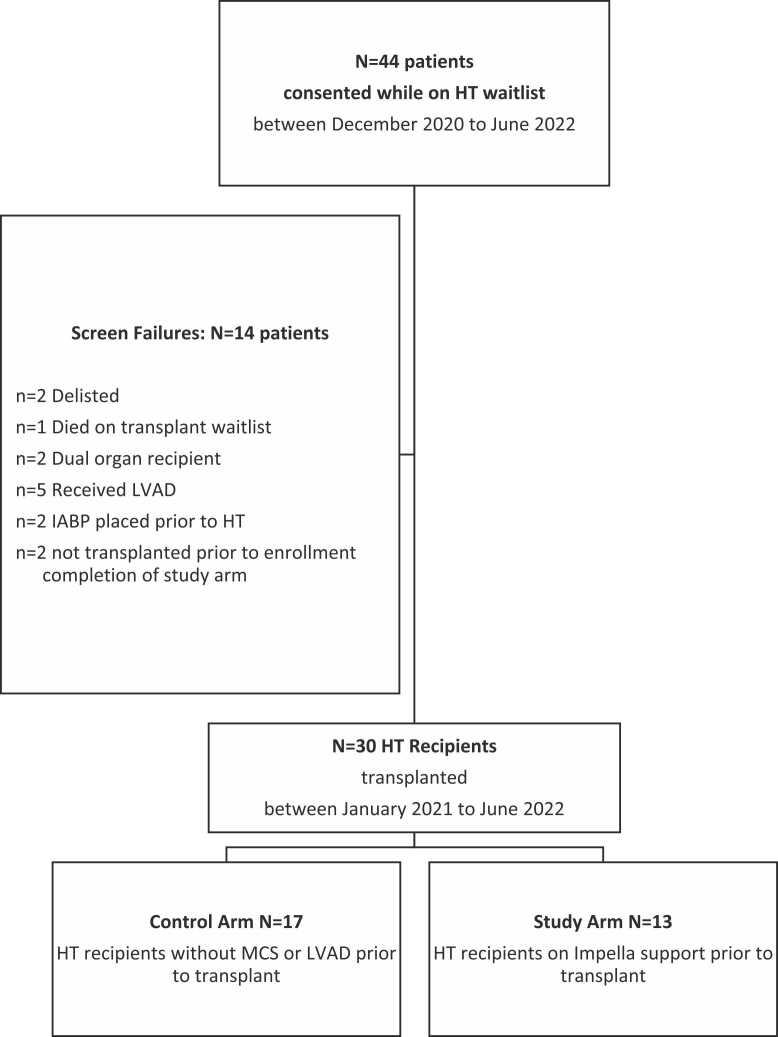
Patient enrollment flow diagram. HT, heart transplantation; IABP, Intra-aortic Balloon Pump; LVAD, left ventricular assist device; MCS, mechanical circulatory support.

Of 44 patients consented, 13 HT recipients were included on Impella support, 17 HT recipients without mechanical circulatory support as controls, and 14 screen-failed due to meeting exclusionary criteria prior to HT (*n* = 12) or not transplanted prior to completion of enrollment in study arm (*n* = 2) (Figure 1). Baseline characteristics, reported in Table 1, did not differ significantly between groups (median age 59 years, 77% male). Patients in the study arm were on Impella support for a median of 7 days [IQR: 5-15] with 12 of 13 patients supported for a duration ≤21 days. All of the patients in the Impella group were United Network for Organ Sharing status 2 with a median time on the waitlist of 5 days while the majority of patients in the control group were United Network for Organ Sharing status 3 (35%) and 4 (59%) with a median time on the waitlist of 53 days. The median number of prior pregnancies among females were 2 [IQR: 1-3].

**Table 1:** Pretransplant Patient Characteristics

	Overall (*n* = 30)	Control (*n* = 17)	Impella (*n* = 13)	*p*-value
*A. Baseline patient characteristics*				
Age (years)	59 [52, 66]	59 [52, 63]	59 [53, 66]	0.83
Sex, male	23 (77%)	12 (71%)	11 (85%)	0.43
BMI at transplant (kg/m^2^)	28 [25, 29.8]	27 [25, 29]	28 [25, 30]	0.85
Race				0.70
African American	10 (33%)	5 (29%)	5 (38%)	
White	20 (67%)	12 (71%)	8 (62%)	
Medical history				
Diabetes	18 (60%)	10 (59%)	8 (62%)	1
Hypertension	16 (53%)	11 (65%)	5 (38%)	0.29
Heart failure etiology, ischemic	10 (33%)	6 (35%)	4 (31%)	1
Prior sternotomy	8 (27%)	7 (42%)	1 (8%)	0.09
Prior pregnancy	7 (23%)	5 (29%)	2 (15%)	0.28
*B. Pretransplant characteristics*				
Impella support duration (days)	n/a	n/a	7 [5,15]	n/a
Infections on Impella support	n/a	n/a	1 (8%)	n/a
Use of blood products pre-Impella	n/a	n/a	1 (8%)	n/a
Use of blood products post-Impella/pretransplant	2 (7%)	0 (0%)	2 (15%)	0.18
Induction				0.42
None	26 (87%)	16 (94%)	10 (77%)	
Simulect – basiliximab	2 (7%)	0 (0%)	2 (15%)	
ATG/ATGAM	2 (7%)	1 (6%)	1 (8%)	
Retrospective crossmatch (RXCM)				0.80
Negative	23 (79%)	12 (75%)	11 (84%)	
B cell positive, T cell negative	2 (7%)	1 (6%)	1 (8%)	
B cell positive, T cell positive	4 (14%)	3 (19%)	1 (8%)	
Not done	1 (3%)	1 (6%)	0 (0%)	
DSA at RXCM	6 (20%)	4 (24%)	2 (15%)	0.67
Virtual crossmatch (VXCM)				0.11
Negative	25 (83%)	14 (82%)	11 (85%)	
B cell positive, T cell positive	3 (10%)	3 (18%)	0 (0%)	
B cell positive, T cell negative	2 (7%)	0 (0%)	2 (15%)	
UNOS status				<0.001
2	14 (47%)	1 (6%)	13 (100%)	
3	6 (20%)	6 (35%)	0 (0%)	
4	10 (33%)	10 (59%)	0 (0%)	
Inotropes at home	6 (20%)	6 (35%)	0 (0%)	0.02
DCD donor	1 (3%)	0 (0%)	1 (8%)	0.43
Hepatitis-C positive donor	1 (3%)	1 (6%)	0 (0%)	1.00
Time on waitlist (days)	14.5 [5, 76.3]	53 [20, 189]	5 [4, 12]	<0.001

Abbreviations: ATG/ATGAM, anti-thymocyte globulin; BMI, body mass index; cPRA, calculated panel reactive antibodies; DCD, donor after circulatory death; HLA, human leukocyte antigen; RXCM, retrospective crossmatch; UNOS, United Network for Organ Sharing; VXCM, virtual crossmatch.

### Pretransplant HLA antibody assessments

**Figure 2:**
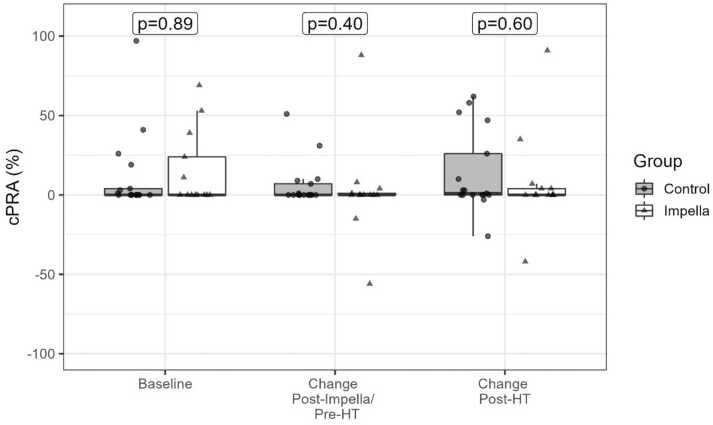
Change from baseline post-Impella (peak cPRA – baseline cPRA) and change postheart transplant (peak cPRA – baseline cPRA) comparing Impella patients to controls. cPRA, calculated panel reactive antibodies; HT, heart transplantation.

Baseline pretransplant HLA antibodies were observed in 7 of 17 and 5 of 13 patients in the control and Impella groups, respectively, with both class I (37%) and class II (13%) antibodies present. Pretransplant HLA assessments are compared in Table 2A. Median cPRA of 0% at baseline and 2.5% at peak pre-HT observed in both groups did not differ significantly (*p* = 0.89 and *p* = 0.71, respectively). At the peak level post-Impella and pretransplant, 3 additional Impella patients and 2 additional control patients developed HLA antibodies with no significant difference in the prevalence between groups (9 of 17 and 8 of 13, *p* = 0.72). The median change from baseline of 0% [IQR: 0%-3.2%] did not differ significantly between groups (*p* = 0.40, Figure 2). Following adjustment for sex, infection, and baseline cPRA, there was still no significant difference between groups (*p* = 0.98).

**Table 2:** HLA Assessments at Baseline (Pre-Impella and Pretransplant), Peak Pretransplant, and Peak Post-Transplant

	Overall (*n* = 30)	Control (*n* = 17)	Impella (*n* = 13)	*p*-value
*A. Pretransplant HLA assessment*				
cPRA				
Baseline	0 [0, 17]	0 [0, 4]	0 [0, 24]	0.89
Peak Post-Impella	2.5 [0, 31.8]	2 [0, 31]	4 [0, 32]	0.71
Change from baseline	0 [0, 3.2]	0 [0, 7]	0 [0, 1]	0.40
Positive HLAs				
Baseline	12 (40%)	7 (41%)	5 (38%)	1.00
Class I	11 (37%)	7 (41%)	4 (31%)	0.71
Class II	4 (13%)	2 (12%)	2 (15%)	1.00
Peak	17 (57%)	9 (53%)	8 (62%)	0.72
Class I	15 (50%)	9 (53%)	6 (46%)	1.00
Class II	5 (17%)	2 (12%)	3 (23%)	0.63
*B. Post-transplant HLA assessment*				
cPRA				
Peak post-transplant	2 [0, 49.8]	1 [0, 52]	7 [0, 43]	0.93
Change from baseline	0 [0, 9.2]	1 [0, 26]	0 [0, 4]	0.60
Positive HLAs	17 (57%)	10 (59%)	7 (54%)	1.00
Class I	14 (47%)	9 (53%)	6 (46%)	1.00
Class II	8 (27%)	5 (29%)	3 (23%)	1.00
Positive DSAs	10 (33%)	6 (35%)	4 (31%)	1.00
C1Q-positive DSA	2 (7%)	2 (12%)	0 (0%)	0.40
Maximum MFI				1.00
Low (<4,000)	5 (17%)	3 (18%)	2 (15%)	
Moderate (4,000-9,999)	4 (13%)	2 (12%)	2 (15%)	
High (>10,000)	1 (3%)	1 (6%)	0 (0%)	
C1Q positive DSA	2 (7%)	2 (12%)	0 (0%)	0.49

Abbreviations: C1Q, complement component 1q; cPRA, calculated panel reactive antibodies; DSA, donor-specific antibodies; HLA, human leukocyte antigen; MFI, mean fluorescence intensity.

Baseline, peak, and change from baseline were compared between Impella and control groups.

### Post-transplant HLA antibody assessments

Post-transplant HLA assessments are compared in Table 2B. The prevalence of HLA antibodies were 10 of 17 and 7 of 13 in the control and Impella groups, respectively, where relative to preheart transplant results there was 1 additional Impella patient with HLAs, 4 additional control patients with HLAs, and 2 Impella and 3 control patients where cPRA returned to 0%. There was no significant difference between groups in peak cPRA post-transplant (median 1% vs 7%, *p* = 0.93) or in cPRA change from baseline (median 1% vs 0%, *p* = 0.60*,* Figure 2). There remained no significant difference between groups following adjustment for sex, infection, and baseline cPRA (*p* = 0.57). Moreover, the prevalence in DSAs (35% vs 31%, *p* = 1.00) and strength at the maximum recorded MFI did not differ significantly between groups (*p* = 1.00).

### Outcomes at 1 year post-transplantation

One patient in the Impella group was admitted for acute cellular rejection and required treatment. No antibody-mediated rejection events occurred in either group. Additionally, 1 Impella patient died during the index transplant admission due to infection post-transplant and 1 control patient due to primary graft dysfunction. Post-transplant outcomes within 1 year were similar between groups with respect to treatment of infection, rejection, hospitalization, and development of cardiac allograft vasculopathy (Table 3).

**Table 3:** Outcomes at 1-Year Post-Transplant

	Overall (*n* = 30)	Control (*n* = 17)	Impella (*n* = 13)	*p*-value
Length of stay post-transplant (days)	13 [9, 17]	11 [8, 16]	15 [11, 22]	0.19
Vital status, deceased	2 (7%)	1 (6%)	1 (9%)	1
Primary graft dysfunction	1 (4%)	1 (6%)	0 (0%)	1
DSA present	10 (30%)	6 (35%)	4 (31%)	1
Treated ACR	1 (3%)	0 (0%)	1 (9%)	0.43
Infection requiring hospitalization and/or intravenous antibiotics	12 (40%)	7 (41%)	5 (38%)	1
Treated cytomegalovirus	11 (37%)	6 (35%)	5 (39%)	1
Noninfection related hospitalization	15 (5%)	9 (53%)	6 (46%)	1
Chronic kidney disease, Class at 1 year				0.43
1	3 (12%)	1 (6%)	2 (16.7%)	
2	8 (31%)	7 (44%)	2 (16.7%)	
3A	10 (39%)	5 (31%)	6 (50%)	
3B	3 (12%)	1 (6%)	2 (16.7%)	
4	1 (4%)	1 (6%)	0 (0%)	
5	1 (4%)	1 (6%)	0 (0%)	
Malignancy	3 (11%)	3 (19%)	0 (0%)	0.25
Cardiac allograft vasculopathy >0 on angiography	5 (19%)	3 (20%)	2 (17%)	1

Abbreviations: ACR, acute cellular rejection (Grade ≥2R); DSA, donor-specific antibodies.

### Patients with de novo HLAs

In the 8 patients (4 Impella and 4 control) developed de novo HLAs post-Impella or post-HT that had cPRA of 0% at baseline, timing of peak HLA measurements are summarized in Table 4. In 3 patients (1 Impella and 2 controls) the de novo HLAs were donor specific. All of the patients were male. No blood products were used in any of the patients during the study period and none of the patients had treated or biopsy-proven antibody-mediated rejection. One patient in the Impella group had acute cellular rejection 255 days post-transplant. Peak cPRA post-HT ranged between 1% and 91%. Only one patient, supported with Impella for 48 days, had persistent HLAs for the entire 1-year follow-up post-HT.

**Table 4:** Summary of HLAs, Treated Rejection, and Treated Infections in Patients That Developed HLAs Post-Impella or Post-Transplant in Whom Baseline cPRA Was 0%

Patient	cPRA peak post-Impella/pre-HT (days post-Impella)	cPRA peak post-HT (days post HT)	HLA antibody class	Treated rejection (days post-HT)	Treated infection (days post-HT)
*Impella Patients (n* *=* *4 of 13)*					
1	1%, 0 days	0%, 358 days	Class 1	No	*Enterococcus* urinary tract infection, 8 days
2	0%, 4 days	7%, 244 days	Class 1	ACR, 225 days	No
3	4%, 0 days	0%, 364 days	Class 1	No	Vancomycin-resistant *Enterococcus* chest tube infection, 50 days
4	88%, 28 days	91%, 130 days	Class 1 and 2	No	No
*Control Patients (n* *=* *4 of 17)*					
5	n/a	1%, 277 days	Class 1	No	CMV infection, 277 days
6	n/a	52%, 43 days	Class 1 and 2	No	No
7	n/a	3%, 46 days	Class 1	No	CMV and EBV viremia, 46 days
8	n/a	58%, 336 days	Class 2	No	CMV viremia, 166 days
					Lyme disease, 291 days

Abbreviations: EBV, Epstein-Barr virus; CMV, cytomegalovirus; cPRA, calculated panel reactive antibodies; HLA, human leukocyte antigen; HT, heart transplantation.

### Blood products

**Figure 3:**
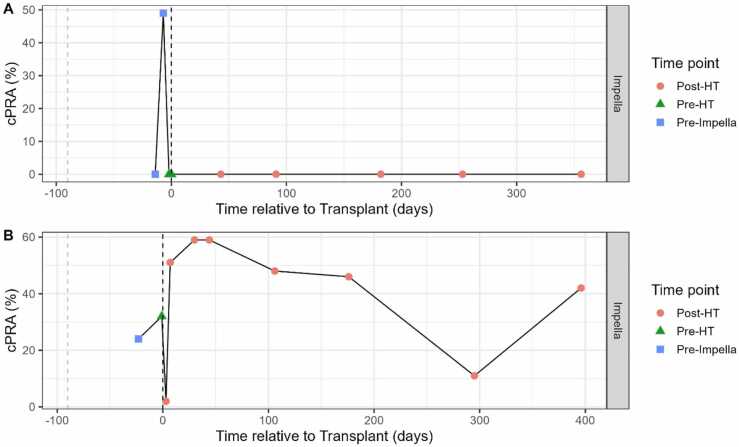
HLA profiles in 2 Impella patients with PRBCs at Impella insertion. (A) PRBCs were used 8 days prior to Impella insertion and at Impella insertion. cPRA returned to 0% post-Impella insertion and remained 0% post-HT. (B) Two units of PRBCs were used at Impella insertion. cPRA increased post-transplantation. cPRA, calculated panel reactive antibodies; HT, heart transplantation; HLA, human leukocyte antigen; PRBCs, packed red blood cells.

For the control arm, there were no patients that required blood products within 6 months of transplantation. Blood products were used in 2 Impella patients prior to transplant. In the first patient, 1 unit of packed red blood cells (PRBCs) was used 8 days prior to Impella implant and a second unit of PRBCs at Impella insertion. This patient’s cPRA increased from 0% at 1 day prior to the first unit of PRBCs to 49% 6 days following (and 2 days prior to Impella) PRBC use, then returned to 0% following Impella implant and remained constant up to 1-year post-transplant (Figure 3A). In the second patient, prior to Impella implant and the use of 2 units of PRBCs, cPRA was 24% and increased post-transplant to a peak of 59% (Figure 3B).

## Discussion

We prospectively investigated the incidence of allosensitization in Impella 5.5 patients awaiting HT and effects on post-transplant outcomes in the current heart allocation system. We demonstrated no significant differences between Impella 5.5 patients and HT-candidate controls regarding the increase in de novo HLA antibodies nor post-transplant morbidity outcomes, including hospitalization duration, rehospitalization, treatment for rejection, and post-transplant 1-year mortality.

Along with well-known sensitizing factors, such as high baseline cPRA, female sex, prior transplantation, transfusion, pregnancy, and infection, durable LVADs are now considered a significant and independent risk factor for allosensitization.^8^, ^9^, ^10^, ^11^ Although varying hypotheses have been generated to explain this phenomenon, convincing evidence remains lacking. A popular hypothesis is that immune-mediated HLA antibody formation is triggered at the time of transfusion when blood is in contact with the neointimalized surfaces of these devices.^12^, ^13^, ^14^ The device biomaterials, namely the polyurethane diaphragm and polytetrafluoroethylene components of older generation LVADs have been proposed as the mechanism responsible for the increased immunologic response.^12^ Colonization of durable LVAD surfaces with T cells, macrophages, and monocytes have been argued as supporting evidence of this hypothesis. In 6 out of 15 patients with a durable ventricular assist device, the platelet activation pattern exhibits continual activation while maintaining reactivity, which is linked to a sustained heightened inflammatory condition and endothelial activation.^15^ In our study, only 1 out of 13 patients showed increased level of HLA antibodies post-Impella which may reflect the role of the persistence of activation in temporary devices in induction of HLA allosensitization. The adherence of T cells to this “pseudo-intima” triggers aberrant T-cell activation with subsequent proliferation, which is followed by B-cell activation mediated through CD40-CD40 ligand interactions and anti-HLA antibody formation.^2^, ^12^ To date, there are no contemporary studies evaluating potential mechanisms of allosensitization in the newer generation of durable LVADs (namely, HeartMate 3 (Abbott, Chicago, IL) and Evaheart (Evaheart, Houston, TX)), nor Impella, given removal or lower biologic membrane and textured surface area when compared to historical LVADs.

In our study, only 2 Impella patients received 1 unit of blood product prior to HT and prior studies have shown that the risk of sensitization with mechanical circulatory support devices appears to be independent of red blood cell transfusions.^15^ This low rate of transfusion minimized the effect on the HLA antibody development. Furthermore, pretransplant sex and history of pregnancy were comparable between both groups.

De novo HLA antibodies could develop if sufficient time is provided for new intima formation and subsequent immune cell activation as seen in patients with LVAD.^16^ However despite contemporary studies reporting allosensitization during placement of durable LVADs, it is far less likely for neointimalization and immune cell adhesion to occur with our study given median duration of 7 days support with Impella 5.5. Only 1 patient in the absence of other sensitizing events was supported for an Impella for 48 days with persistent HLAs 1 year post-HT without rejection or hospitalizations, supporting prior retrospective analysis that sensitization may in fact become more prevalent with simply with increasing the length of support.^3^, ^15^ Thus, the overall results of our study are not surprising, however warrant specific areas of investigation such as examining the relationship between longer duration and role of device-neointimalized surfaces in HLA antibody development in patients with the Impella 5.5.

## Limitations

Limitations of our study include a small sample size from a single center that could be underpowered to detect smaller differences and may not be generalizable to other centers. However, the sample size provided sufficient power to detect differences in cPRA of 16% ± 16% between groups. Larger studies are needed to confirm our results and be representative of a more diverse demographic population, including more females. Furthermore, this is the only known prospective study reported to date evaluating the use of Impella 5.5 in this particular clinical population in the United States that currently comprises 4% of heart transplants and is continuing to rise along with a notable increase in waitlist times, now well beyond 14 days.^17^, ^18^ Additionally, more than 20% of transplant recipients are listed as Status 2 at time of transplant with 40% of these patients being listed by exception criteria.^19^, ^20^ Thus, tMCS is being utilized at an exponential rate as bridge to HT with shorter durations to transplant than those without a device, albeit device time to transplant is now exceeding 14 days as well. Our data are clinically impactful as we did not observe a significant difference between groups in HLA development or post-transplant outcomes. Larger studies, especially as the 2018 heart allocation system reaches equilibrium, are warranted to study the relationship of prolonged device duration and immune-related events in the peri- and post-transplant period.

## Conclusion

In conclusion, our study suggests that short-term use of Impella 5.5 does not increase risk of de novo HLA antibody development nor increase the risk of allograft rejection in the current 2018 heart allocation system. Larger studies with a mechanistic component to investigate the association of alloimmunization and tMCS devices, such as Impella 5.5, are needed to verify these preliminary findings.

## Disclosure statement

The authors declare the following financial interests/personal relationships which may be considered as potential competing interests: Amit Alam: Speaker for CareDx and Abbott. Medhat Askar: Scientific Advisory Board of CareDx, Thermo Fisher, Immucor and Hansa Biopharma. Shelley Hall: Consultant/Advisor for Abbott, Abiomed, Evaheart, Natera, and CareDx. The remaining authors have nothing to disclose.

Acknowledgments: None.

Cardiovascular Research Review Committee Internal Institutional Grant.
